# Impact of D3 lymph node dissection extent on postoperative bowel function and nutritional status in right-sided colon cancer: a prospective randomized controlled trial

**DOI:** 10.1097/JS9.0000000000003485

**Published:** 2025-09-09

**Authors:** Xinxiu Zhou, Zhou Shang, Feng Tian, Jiyuan Liu, Baolin Zhang, Mingshuai Bai, Guodong Lian, Leping Li, Changqing Jing, Yuezhi Chen

**Affiliations:** a Department of Gastrointestinal Surgery, Shandong Provincial Hospital Affiliated to Shandong First Medical University, Jinan, Shandong, China; b Department of Critical Care Medicine, Shandong Provincial Second People’s Hospital, Jinan, Shandong, China

**Keywords:** D3 lymphadenectomy, nutritional status, postoperative diarrhea, right-side colon cancer, superior mesenteric artery, superior mesenteric vein

## Abstract

**Aim and background:**

This study investigates the impact of D3 lymph node dissection extent on postoperative bowel function and nutritional status in patients undergoing radical surgery for right-sided colon cancer. Given that postoperative diarrhea can significantly affect the quality of life, we examined whether dissection boundaries influence these outcomes.

**Methods:**

This was a prospective, randomized controlled trial conducted at a high-volume tertiary hospital. Eligible patients with right-sided colon cancer were randomly assigned to undergo D3 lymphadenectomy extending to either the left side of the superior mesenteric vein (SMV group) or the left side of the superior mesenteric artery (SMA group). The primary outcome was the incidence of postoperative diarrhea. Secondary outcomes included chylous leakage, number of retrieved lymph nodes, postoperative recovery parameters, and nutritional status.

**Results:**

A total of 81 patients were enrolled, and 76 patients (SMV: 38, SMA: 38) were included in the final analysis. The incidence of postoperative diarrhea was significantly higher in the SMA group than in the SMV group (39.5% vs. 18.4%; *P* = 0.043). Chylous leakage was also more frequent in the SMA group (21.1% vs. 5.3%; *P* = 0.042). Although more lymph nodes were retrieved in the SMA group than in the SMV group (*P* = 0.043), the number of positive lymph nodes did not differ significantly between the groups (*P* = 0.370). Subgroup analysis showed that female patients in the SMA group had a significantly higher incidence of diarrhea compared with those in the SMV group (53.3% vs. 9.1%; *P* = 0.019), while male patients in the SMA group had a higher incidence of chylous leakage (30.4% vs. 7.4%; *P* = 0.035). Patients with stage I–II tumors in the SMA group experienced more postoperative diarrhea than those in the SMV group (50.0% vs. 18.2%; *P* = 0.029), whereas no significant difference was observed in stage III patients (*P* > 0.05). No significant differences in postoperative recovery times or nutritional status were noted between the groups. At a median follow-up of 37 months, the incidence of chronic diarrhea was 21.1% in the SMA group and 10.5% in the SMV group (*P* = 0.208). No significant differences in recurrence or survival were observed between groups.

**Conclusion:**

Extending D3 lymphadenectomy to the SMA increases lymph node yield but is associated with a higher incidence of postoperative diarrhea and chylous leakage, without clear short-term oncologic benefit. These findings underscore the importance of balancing oncologic thoroughness with functional recovery and may inform more individualized, patient-centered surgical planning through shared decision-making.

## Background

Complete mesocolic excision (CME) with D3 lymphadenectomy is a key surgical approach for right-sided colon cancer, emphasizing high ligation of the supplying blood vessels and meticulous mesenteric resection to improve oncologic outcomes^[1,2]^. However, the optimal medial boundary of D3 lymphadenectomy remains controversial^[3,4]^. While several retrospective studies and experts advocate limiting dissection to the left margin of the superior mesenteric vein (SMV)^[5,6]^, the Japanese Society for Cancer of the Colon and Rectum guidelines recommend extending it to include lymph nodes around the superior mesenteric artery (SMA), necessitating SMA exposure^[7]^.

**HIGHLIGHTS**

- Extending D3 lymphadenectomy to the SMA increases node yield but shows no clear oncologic advantage over SMV dissection.
- SMA-level dissection carries higher risks of postoperative diarrhea and chylous leakage, requiring careful risk–benefit assessment.
- Short-term nutritional status was preserved, but long-term functional consequences remain uncertain.
- Findings support shared decision-making, tailoring lymphadenectomy extent to balance oncologic benefit with quality of life.

Multiple retrospective studies have shown that extending lymphadenectomy to the SMA increases the total number of retrieved lymph nodes but does not necessarily yield more positive nodes^[8-10]^. Furthermore, evidence from retrospective and anatomical studies suggests that lymph node dissection guided by the SMA may increase the risk of postoperative gastrointestinal dysfunction, including diarrhea and chylous leakage, likely due to disruption of the dense lymphatic and neural networks surrounding the artery^[11,12]^. Similar complications have been reported in surgeries involving the pancreas^[13,14]^, mesentery^[15]^, and small bowel transplantation^[16,17]^. Severe postoperative diarrhea can impair short-term nutritional status, reduce quality of life, delay chemotherapy, and ultimately impact long-term prognosis^[18,19]^.

This study aimed to compare postoperative bowel function, diarrhea incidence, and nutritional status between these two approaches in patients undergoing radical surgery for right-sided colon cancer. This work complies with the TITAN 2025 guideline for transparency in reporting the use of artificial intelligence in research and writing processes^[20]^.

## Methods

### Trial design

This study is a single-center, open-label, prospective randomized controlled trial comparing the effects of different extents of D3 lymphadenectomy (SMA vs. SMV) on postoperative bowel function and nutritional status in patients undergoing elective right hemicolectomy for right-sided colon cancer. Patients were randomly assigned to one of two groups: the SMV group, where D3 lymphadenectomy extended to the left margin of the SMV, and the SMA group, where it extended to the left margin of the SMA. All participants provided informed consent before enrollment. The work has been reported in line with Consolidated Standards of Reporting Trials (CONSORT) Guidelines^[21]^.

### Inclusion criteria

Participants were adults aged 18–75 years with histologically confirmed right-sided colon cancer based on preoperative colonoscopic biopsy. And based on CT and colonoscopy, the differences in tumor location were identified (cecum, ascending colon, hepatic flexure, or right transverse colon).

### Exclusion criteria

Exclusion criteria included patients with multiple primary colon cancers, colonic obstruction or perforation due to tumor, distant metastases (e.g., liver or lung) on CT/MRI, history of abdominal surgery with dense intra-abdominal adhesions, severe cardiopulmonary disease contraindicating surgery, long-term steroid use, or an ASA score of IV or V.

### Randomization

Patients meeting the eligibility criteria were randomly assigned in a 1:1 ratio to either the SMA or SMV group using a computer-generated randomization sequence to better balance the statistical variables between the two groups, such as age, sex, smoking and alcohol use, surgical approach, and ASA grade. Allocation concealment was maintained using sequentially numbered, opaque, sealed envelopes prepared by an independent researcher not involved in the study. The randomization assignment was revealed to the surgical team only after patient enrollment to prevent selection bias.

### Surgical procedure

All surgeries were performed under standardized general anesthesia using propofol, cisatracurium besilate, etomidate, sevoflurane, and sufentanil. Ondansetron (4 mg) was administered intravenously at the end of surgery to prevent nausea. Cefuroxime (1.5 g) was given intravenously 30 minutes before incision. A single experienced surgeon performed all procedures to minimize variability. Surgery was conducted using laparoscopic or robotic techniques, with standardized CME and D3 lymphadenectomy. The key difference between groups was the extent of lymph node dissection:

SMV group: Lymphadenectomy included nodes to the left of the SMV.

**Figure 1.**
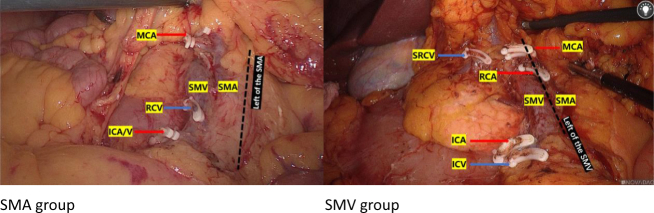
Surgical operation diagram for the SMV group and the SMA group.

SMA group: Lymphadenectomy included nodes to the left of the SMA (Fig. 1).

### Outcome measures

The primary outcome was the incidence of diarrhea, while secondary outcomes included the incidence of chylous leakage, length of hospital stay (LOS), postoperative complications such as surgical site infections and anastomotic leakage, time to first flatus, time to tolerance of a liquid diet, total lymph node count, number of positive lymph nodes, and nutritional markers such as hemoglobin (HB), pre-albumin (PA), albumin (ALB), and retinol-binding protein (RBP).

Diarrhea was defined as more than three watery stools per day, with at least one episode exceeding 50 mL, occurring within 30 days after surgery. Chylous leakage was identified by a triglyceride level of ≥110 mg/dL (1.2 mmol/L) in abdominal drainage following a meal.

### Sample size

Based on retrospective data, the expected diarrhea incidence post-resection was 15%. It was hypothesized that SMA lymph node dissection could increase diarrhea incidence to 45%. With a 5% two-tailed type I error rate and 80% power, a sample size of 33 patients per group was calculated, with no allowance for loss to follow-up.

### Statistical analysis

Categorical variables are presented as numbers and analyzed using a χ^2^ or Fisher’s exact tests, as appropriate. Normally distributed continuous variables were reported as mean ± standard deviation, and an independent sample *t*-test was used to compare the differences between the experimental and control groups. Non-normally distributed continuous variables were reported as median and interquartile range (IQR), and non-parametric rank-sum tests were used for the statistical analyses. Data analyses and descriptive statistics were performed using SPSS (version 26.0, IBM Corp., Armonk, NY, USA). Except when specified otherwise, all reported *P* values were two-tailed, with statistical significance set at *P* < 0.05.

## Results

**Figure 2.**
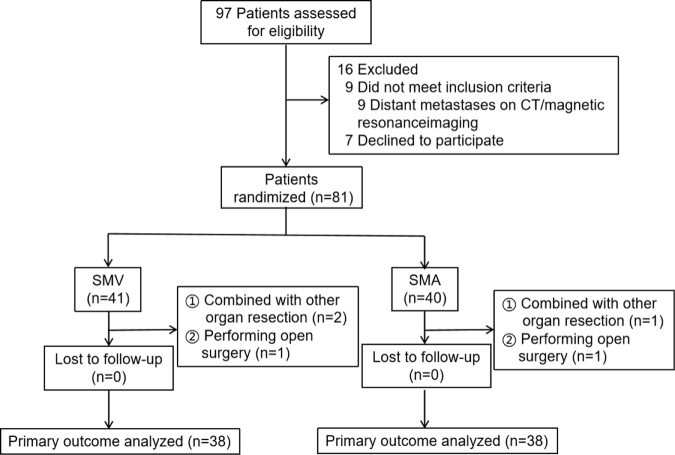
Flow chart of patient selection.

Of the 97 patients who met the inclusion criteria, 9 were excluded due to distant metastasis, and 7 refused to participate. This left 81 eligible patients, randomized into the SMV group (*n* = 41) and the SMA group (*n* = 40). In the SMV group, 2 patients were excluded due to a combined organ resection, and 1 underwent open surgery, resulting in 38 patients. Similarly, in the SMA group, 1 patient was excluded due to combined organ resection, and 1 underwent open surgery, also leaving 38 patients. No patients were lost to follow-up (Fig. 2).

### Patient characteristics

There were no significant differences between the SMV and SMA groups in demographic factors, including sex, age, body mass index (BMI), or nutritional status as assessed by the Nutritional Risk Screening 2002 (NRS-2002) score^[22]^. More than half of the participants were male. Additionally, tumor characteristics were comparable between the two groups, with no statistically significant differences. Tumor location (cecum, ascending colon, hepatic flexure, or right transverse colon) was determined based on colonoscopic findings and intraoperative confirmation. Although not used as a stratification factor during randomization, the distribution of tumor sites was similar between groups (Table 1).

**Table 1:** Demographic and clinical information of patients undergoing SMV and SMA surgery

	SMV	SMA	*P* value
	*n* = 38 (%)	*n* = 38 (%)	
Age (year, mean ± SD)	60 ± 11.6	59 ± 15.1	0.919
Male	27 (71.1)	23 (60.5)	0.333
BMI (mean ± SD)	25.5 ± 4.1	24.4 ± 3.1	0.176
NRS 2002 Score			0.639
<3	16 (42.1)	14 (36.8)	
≥3	22 (57.9)	24 (63.2)	
ASA Score			0.324
II	28 (73.7)	24 (63.2)	
III	10 (26.3)	14 (36.8)	
Hypertension	14 (36.8)	15 (39.5)	0.813
Diabetes	7 (18.4)	8 (21.1)	0.773
Chronic astriction	0	0	1.000
Diarrhea	0	0	1.000
Surgical approach			0.464
Laparoscopy	11 (28.9)	14 (36.8)	
Robotic	27 (71.1)	24 (63.2)	
Tumor site			0.439
Cecum	17 (44.7)	12 (31.6)	
Ascending colon	12 (31.6)	11 (28.9)	
Right flexure	7 (18.4)	13 (34.2)	
Right transverse colon	2 (5.3)	2 (5.3)	
Cancer stage			0.225
I	11(28.9)	5(13.2)	
II	11(28.9)	15(39.5)	
III	16(42.2)	18(47.3)	

SD, standard deviation; BMI, body mass index; NRS, National Risk Screening; ASA, American Society of Anesthesiologists.

### Primary and secondary outcomes

Diarrhea occurred in 22 patients – 7 in the SMV group and 15 in the SMA group – resulting in an overall incidence of 28.9%. The SMV group had a significantly lower incidence of diarrhea compared to the SMA group (18.4% vs. 39.5%, *P* = 0.043; Table 2).

**Table 2:** Outcomes of patients undergoing SMV and SMA surgery

	SMV	SMA	*P* value
	*n* = 38 (%)	*n* = 38 (%)	
Diarrhea	7 (18.4)	15 (39.5)	0.043
Chylous leakage	2 (5.3)	8 (21.1)	0.042
LOS (d, median [IQR])	9 [8,12]	9 [8,11]	0.682
Postoperative hospital stay (d, median [IQR])	5[5, 5]	5[5, 7]	0.169
Wound infection	2(5.3)	3(7.9)	0.644
Anastomotic leakage	0(0.0)	2(5.3)	0.152
Time of first flatus (h, median [IQR])	39.9[36.4, 46.5]	38.0[35.0, 43.3]	0.044
Time to tolerate of liquid diet (h, mean ± SD)	31.3 ± 3.1	30.0 ± 3.1	0.073
Total harvested LNs, (mean ± SD)	20.1 ± 7.3	23.3 ± 6.0	0.043
Number of patient with positive LNs	14(36.8)	15(39.5)	0.813
Positive harvested LNs (median [IQR])	2.5[1, 5]	2.2[1,2]	0.370
Surgical time (median [IQR], min)	218[184, 240]	221[195, 235]	0.839
Blood loss (median [IQR], mL)	56[34, 96]	53[35, 65]	0.708

LOS, length of stay; IQR, interquartile range; SD, standard deviation; LNs:lymph nodes.

Although the SMA group had a significantly higher total lymph node yield than the SMV group (*P* = 0.043, Table 2), the number of positive lymph nodes among patients with metastases did not differ significantly between groups (*P* = 0.370, Table 2).

The incidence of chylous leakage was significantly higher in the SMA group than in the SMV group (21.1% vs. 5.3%, *P* = 0.042). Median hospital length of stay was 9 days in both groups, with a median postoperative stay of 5 days. Abdominal distension within the first 24 hours post-surgery was comparable between groups. Although the SMA group showed a trend toward earlier tolerance of a liquid diet, this difference was not statistically significant (*P* = 0.073, Table 1). However, time to first flatus was significantly shorter in the SMA group (*P* = 0.044, Table 1).

**Figure 3.**
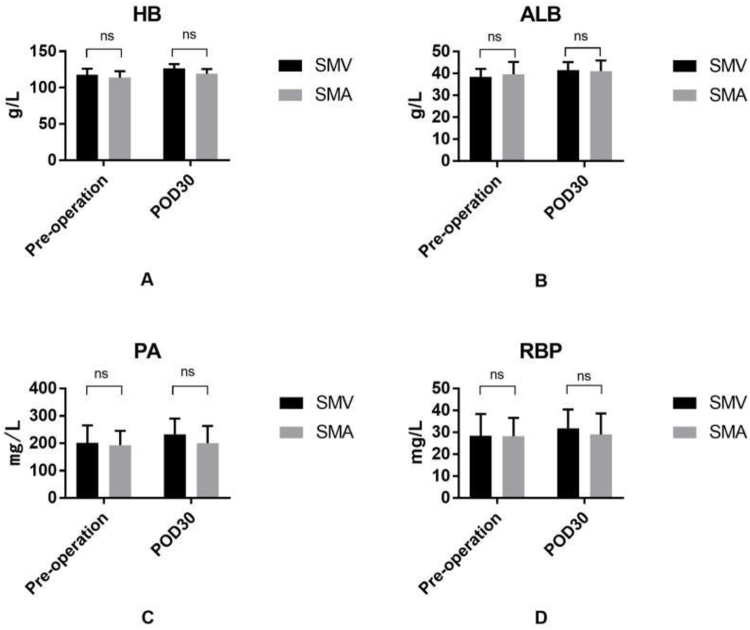
Comparison of preoperative and postoperative nutritional indicators between SMV and SMA groups. Nutritional parameters were measured preoperatively and on postoperative day 30 (POD30) in patients who underwent lymphadenectomy to either the SMV or SMA. Data are presented as means with standard deviations. Between-group comparisons at each time point were conducted using the independent-sample t-test (SPSS, version 26.0). No statistically significant differences were observed (ns: not significant).

No significant differences were observed in nutritional indicators between the groups (Fig. 3), and other secondary outcomes were also comparable.

### Subgroup analysis

**Figure 4.**
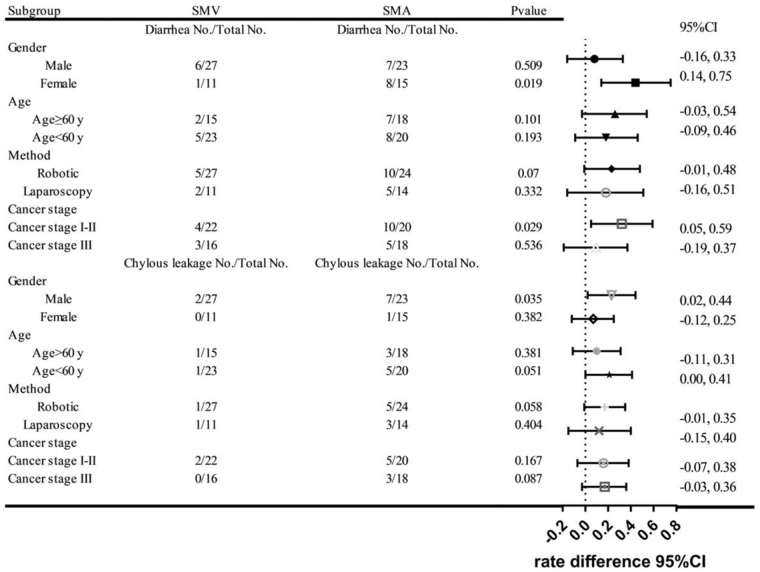
Subgroup analysis of postoperative diarrhea and chylous leakage according to lymphadenectomy extent (SMV vs. SMA). Data are presented as a forest plot, with central markers indicating rate differences and horizontal lines representing 95% confidence intervals. Subgroup analyses were stratified by sex, age, surgical approach, and cancer stage. Positive rate differences indicate higher complication rates in the SMA group compared to the SMV group.

Furthermore, subgroup analysis revealed that male patients in the SMA group experienced a significantly higher incidence of chylous leakage compared to the SMV group (30.4% vs. 7.4%, *P* = 0.035). In contrast, female patients in the SMA group had a significantly higher incidence of diarrhea compared to the SMV group (53.3% vs. 9.1%, *P* = 0.019). In patients with stage I-II tumors, the incidence of diarrhea was significantly higher in the SMA group compared to the SMV group (18.2% vs. 50%, *P* = 0.029). However, in patients with stage III tumors, no significant difference in diarrhea incidence was observed between the two groups (*P* > 0.05). The incidence of chylous leakage did not significantly differ between the SMV and SMA groups across all tumor stages (*P* > 0.05) (Fig. 4).

In addition, there was no difference in the incidence of diarrhea and chyle leakage among patients aged over 60 or under 60 years old (*P* > 0.05). Similarly, among all patients, regardless of whether they underwent robotic or laparoscopic surgery, there was no difference in the incidence of diarrhea and chyle leakage (*P* > 0.05) (Fig. 4).

### Long-term oncologic and functional outcomes

As of the latest follow-up on 31 May 2025 (median follow-up: 37 months), four patients in the SMA group and four4 in the SMV group developed distant metastases. In the SMA group, metastases included liver (*n* = 2), peritoneum (*n* = 1), and combined peritoneal and ovarian spread (*n* = 1). In the SMV group, metastases were to the liver (*n* = 2), peritoneum (*n* = 1), and thoracic spine (*n* = 1). Six patients in the SMA group died during the follow-up period, four due to tumor recurrence, one due to cardiopulmonary disease, and one due to abdominal sepsis from chylous leakage. In contrast, two deaths occurred in the SMV group: one from tumor progression and one from immunosuppression-associated pulmonary tuberculosis. Median OS and DFS were not reached in either group at the time of analysis.

At 1-year follow-up, chronic diarrhea – defined as ≥3 loose or watery stools per day for at least 4 consecutive weeks – was observed in four patients (10.5%) in the SMV group and eight patients (21.1%) in the SMA group. There was no statistically significant difference between the groups (*P* = 0.208).

## Discussion

Current guidelines from the European Society for Medical Oncology^[23,24]^, the National Comprehensive Cancer Network^[25–27]^, and the Chinese Society of Clinical Oncology^[28,29]^ acknowledge that D3 lymph nodes are located at the root of major blood vessels. However, they do not clearly define the medial boundary for lymphadenectomy in right-sided colon cancer^[30,31]^. The optimal extent of D3 lymph node dissection remains debated, with traditional surgical approaches prioritizing extensive lymphatic clearance while often overlooking its potential impact on postoperative bowel function^[32,33]^. This randomized controlled trial compared the effects of D3 lymphadenectomy extending to the left margin of the SMA versus the SMV on postoperative bowel function and nutritional status.

Our findings indicate that SMA-oriented dissection is associated with a significantly higher incidence of postoperative diarrhea and chylous leakage compared to SMV-oriented dissection. Although SMA dissection resulted in a greater number of harvested lymph nodes, it did not increase the detection of positive lymph nodes.

The higher incidence of diarrhea in the SMA group may be attributed to nerve and lymphatic damage. This is consistent with previous reports suggesting that more extensive lymphadenectomy in this region increases the risk of bowel dysfunction due to nerve disruption^[11]^. The resultant altered bowel motility – characterized by increased peristalsis and reduced contractility – may take up to 6 months to normalize. Postoperative diarrhea can significantly affect patient recovery, leading to prolonged rehabilitation and delayed initiation of adjuvant therapies^[34]^. Therefore, it is still necessary to carefully evaluate how to develop a surgical plan and determine the optimal range of D3 lymph node dissection before surgery. The observed trend toward a shorter time to first flatus in the SMA group may reflect increased bowel irritation, though further research is needed to confirm this association.

Despite the higher lymph node yield in the SMA group, the number of positive nodes did not differ significantly between groups, suggesting that more extensive lymphadenectomy may not provide additional oncologic benefit. This underscores the need to balance the potential survival advantages of extended lymphadenectomy with the risk of postoperative complications. However, our study lacked long-term follow-up, leaving the impact of extended lymphadenectomy on patient survival uncertain. Future research should evaluate whether the increased node retrieval influences long-term oncologic outcomes.

Chylous leakage, another significant complication, was more frequent in the SMA group. Previous retrospective studies have shown that this may be due to the proximity of the lymphatic vessels to the SMA, which increases the risk of disruption during dissection^[35]^. This complication can be challenging to manage and may prolong hospitalization or require additional interventions, further complicating recovery^[36]^.

Subgroup analysis suggested that the increased risk of diarrhea and chylous leakage in the SMA group may be influenced by both sex and tumor stage. One possible explanation for the higher incidence of diarrhea in female patients undergoing SMA-oriented dissection is their greater susceptibility to autonomic nerve injury. Previous studies have indicated that female autonomic nervous function is more sensitive to damage, which could make them more prone to bowel dysfunction following extensive lymphadenectomy. Additionally, sex-related differences in gastrointestinal motility regulation may contribute to these findings. Estrogen has been implicated in modulating intestinal peristalsis, and its influence on postoperative bowel function may partially explain the increased incidence of diarrhea observed in female patients after SMA-oriented dissection^[37,38]^. However, it is important to acknowledge that direct evidence supporting the hypothesis that the female autonomic nervous system is inherently more vulnerable to surgical injury remains limited. Further research is needed to clarify the underlying mechanisms and determine whether sex-based physiological differences significantly contribute to postoperative bowel dysfunction in patients undergoing SMA-oriented dissection.

Conversely, male patients had a significantly higher incidence of chylous leakage, which may be attributed to greater visceral fat accumulation compared to females^[39]^. This fat accumulation may make it more challenging to identify and preserve lymphatic vessels during surgery, thereby increasing the risk of postoperative complications in colorectal surgery^[40,41]^.

Interestingly, despite extensive lymph node metastasis in stage III patients, our study did not find a significantly higher incidence of postoperative diarrhea in this group following SMA-oriented dissection. This suggests that preoperative tumor invasion of autonomic nerves may not necessarily translate into increased postoperative bowel dysfunction. One possible explanation is that the autonomic nervous system has a certain degree of compensatory capacity, allowing III-stage patients to tolerate additional surgical nerve injury without a significant increase in diarrhea incidence. Additionally, chronic inflammation associated with advanced-stage disease may modulate the gut-neural axis, reducing the sensitivity of the enteric nervous system to postoperative perturbations. Moreover, these patients may have undergone gradual gastrointestinal adaptation due to prolonged tumor progression, allowing better adjustment to postoperative functional changes. Further research is needed to clarify the exact mechanisms underlying these observations.

These findings suggest that the mechanisms underlying postoperative diarrhea and chylous leakage following SMA-oriented dissection are multifactorial, involving both sex-specific physiological differences and tumor-related autonomic nerve involvement. However, these subgroup analyses were exploratory and not prespecified in our study protocol. Given the relatively small sample size, they are underpowered and should be interpreted with caution. Further studies are warranted to validate these findings, which may help guide more individualized surgical strategies for right-sided colon cancer.

Interestingly, despite the higher rates of diarrhea and chylous leakage, no significant differences in short-term nutritional status were observed between groups. This suggests that these complications may not severely impact early postoperative nutritional outcomes. Previous studies have indicated that postoperative hypoalbuminemia can impair the initiation of adjuvant therapy and negatively influence long-term oncologic outcomes^[42–44]^. Although our results demonstrate comparable short-term nutritional effects between SMA and SMV dissection, laboratory markers such as albumin and prealbumin may still serve as useful indicators for identifying patients at risk of delayed recovery. Persistent gastrointestinal dysfunction, including chronic diarrhea or malabsorption, could compromise long-term nutritional status – a possibility that warrants further investigation in future longitudinal studies.

Although the time to first flatus was statistically shorter in the SMA group, the absolute difference between groups was less than 2 hours. This small margin is unlikely to have a meaningful impact on clinical recovery or discharge planning, and thus, its relevance should be interpreted cautiously. The statistical significance may reflect the sample size rather than the true clinical benefit.

Compared with previous retrospective or observational studies assessing D3 lymphadenectomy extent, our study design offers stronger evidence by minimizing selection bias and standardizing outcome assessment. However, the relatively small sample size and single-center nature of the trial may limit the generalizability of our findings, particularly for subgroup analyses, which were exploratory and underpowered. Larger, multicenter trials are needed to validate these observations in diverse populations.

In addition, the open-label design without blinding of outcome assessors may introduce bias, especially for subjective outcomes. Although objective criteria – such as stool volume for diarrhea and triglyceride levels for chylous leakage – were applied, assessment bias cannot be completely ruled out in borderline cases. Validated tools such as the Diarrhea Assessment Scale may provide a more comprehensive evaluation and will be considered in future studies.

Moreover, although the primary focus of this study was on short-term postoperative outcomes such as bowel function and complications, we have included preliminary long-term follow-up data to enhance clinical relevance. At a median follow-up of 37 months, no significant differences in recurrence or survival patterns were observed between groups, and median overall and disease-free survival have not yet been reached. Continued follow-up is ongoing to clarify whether the extent of D3 lymphadenectomy has any long-term oncologic implications. Likewise, we assessed the incidence of chronic diarrhea at 1 year postoperatively, which showed no statistically significant difference between groups. Nonetheless, due to resource constraints, we were unable to evaluate other potentially meaningful long-term outcomes, such as small bowel bacterial overgrowth, vitamin B12 deficiency, or broader quality-of-life measures. Therefore, while our extended follow-up offers early insights into both oncologic and functional trajectories, further longitudinal studies incorporating comprehensive nutritional, microbial, and patient-reported endpoints are needed to fully elucidate the long-term impact of D3 lymphadenectomy extent.

Lastly, although we observed differences in complication rates related to sex and tumor stage, the mechanisms underlying these associations – such as variations in autonomic nerve distribution or lymphatic drainage – remain speculative. Future studies incorporating functional imaging, nerve mapping, or neurophysiologic assessment may provide mechanistic insights that build upon our clinical findings.

## Conclusion

Extending D3 lymphadenectomy to the SMA increases lymph node yield but significantly raises the risk of postoperative diarrhea and chylous leakage without clear oncologic benefits. These findings highlight the need for a balanced approach when determining the extent of lymph node dissection, emphasizing both oncologic thoroughness and postoperative quality of life in patients undergoing surgery for right-sided colon cancer. Importantly, the results may support more nuanced preoperative discussions, enabling surgeons and patients to engage in shared decision-making and tailor the extent of lymphadenectomy to individual clinical priorities and patient preferences.

## Data Availability

The original contributions presented in the study are included in the article/supplementary material. Further inquiries can be directed to the corresponding author.

## Research registration unique identifying number (UIN)

https://www.clinicaltrials.gov/ – for all human studies – free.

## Statement of translational relevance

The findings of this study highlight the need to balance oncological thoroughness with postoperative quality of life in right-sided colon cancer surgery. While extending D3 lymphadenectomy to the SMA increases lymph node yield, it significantly raises the risk of postoperative diarrhea and chylous leakage without clear oncologic benefits. These results suggest that limiting dissection to the SMV may reduce complications while maintaining adequate lymph node retrieval. Future practice should individualize surgical approaches, considering patient factors such as sex and tumor stage, to optimize outcomes. Further research is needed to evaluate long-term survival implications, ensuring both efficacy and patient well-being in cancer care.
